# The Role of REV-ERB Receptors in Cancer Pathogenesis

**DOI:** 10.3390/ijms24108980

**Published:** 2023-05-19

**Authors:** Georgia Gomatou, Anastasia Karachaliou, Orsalia-Zoi Veloudiou, Alexandra Karvela, Nikolaos Syrigos, Elias Kotteas

**Affiliations:** Oncology Unit, Third Department of Medicine, “Sotiria” General Hospital for Diseases of the Chest, National and Kapodistrian University of Athens, 115 27 Athens, Greece

**Keywords:** REV-ERB receptors, nuclear receptors, transcription factors, cancer, circadian rhythm

## Abstract

REV-ERB receptors are members of the nuclear receptor superfamily of proteins, which act as both intracellular receptors and transcription factors, therefore modulating the expression of target genes. REV-ERBs act as transcription repressors because of their unique structure. Their predominant role involves the control of peripheral circadian rhythmicity by participating in a transcription–translation feedback loop with other major clock genes. Regarding their role in cancer pathogenesis, recent studies in various cancerous tissues have revealed that their expression was downregulated in the majority of the cases. Dysregulation of their expression was also implicated in cancer-associated cachexia. The pharmacological restoration of their effects is feasible with synthetic agonists, which have been explored in preclinical studies but with scarce data. There is a need for further investigation, primarily with mechanistic studies, on the effect of the REV-ERB-induced circadian rhythm deregulation in carcinogenesis and cancer-related systemic effects, such as cachexia, in order to address the potential of relevant therapeutic implications.

## 1. Introduction

REV-ERB alpha (REV-ERBα) and REV-ERB beta (REV-ERBβ) are two unique members of the nuclear receptor (NR) superfamily of proteins [1]. The name REV-ERBα is a truncation of ‘reverse c-erbAa’ since the protein’s encoding gene is located in the strand opposite the human thyroid hormone receptor alpha (*THRA*, also known as *c-erbAα*) [1]. A second, highly homologous receptor was later identified and was named REV-ERBβ owing to its homology with REV-ERBα [1].

The NR group comprises molecules acting as receptors, located within the cells and activated by lipid-soluble signals, and as transcription factors regulating gene expression [2]. Their dual role differentiates them from other transcription factors. Moreover, their ligand-binding sites render those molecules appealing targets for drug design. In the past few decades, the role of NRs in physiology and pathophysiology has been the focus of numerous studies, revealing their crucial function in fundamental cellular processes, including metabolism and proliferation [3]. Regarding cancer pathogenesis, although the involvement of particular NRs, namely, the steroid receptors, is well-established, the role of other members of the family remains elusive [4].

In the present article, we focus on two NR members, REV-ERBα and REV-ERBβ, and explore their role in cancer pathogenesis. The REV-ERB receptors are mostly known for regulating the cell-autonomous circadian rhythm and are implicated in several metabolic pathways [5,6]. Accumulating evidence suggests that the aberration of the cellular circadian clock is a non-negligible contributor to cancer pathogenesis [7,8,9,10]. Elucidating the molecular background of the potential association between circadian rhythm and cancer, especially regarding targetable molecules, is of utmost importance. The aim of this narrative review is to summarize the evidence on the involvement of REV-ERB receptors in the initiation and progression of tumors and discuss their therapeutic potential and related challenges.

## 2. Circadian Rhythm

The circadian clock is an endogenous temporal system conserved in animals, plants, fungi, and bacteria, which produces an oscillation of various physiological processes within a period of 24 h (“circa diem”) [11]. The circadian rhythm provides an evolutionary advantage by regulating the synchronization of physiological and cellular processes with the dark–light cycle and therefore contributing to homeostasis [12]. The circadian rhythmicity is “entrained” by cyclic environmental cues such as light, regular feeding, temperature, and exercise, which are termed zeitgebers [13]. However, this system has the ability to remain self-sustained and operate independently of the zeitgeber signals [13].

The mammalian circadian clock displays a hierarchical organization. The central circadian pacemaker is the suprachiasmatic nucleus (SCN) located in the anterior hypothalamus and composed of approximately 15,000 neurons [14]. The SCN neural cells exhibit circadian rhythmicity in terms of gene expression as well as in the rate of action potential firing [14]. SCN coordinates a network of downstream peripheral clocks within the central nervous system and throughout the body tissues. A finding that profoundly modified the view of circadian clocks is that most peripheral pacemakers are intrinsically independent [11,15].

From the top down, through a pathway that converts the photic signals of the external day into the molecular composition of the internal day, light information is collected by specific photosensitive retinal ganglion cells and then transmitted to the SCN via the retinohypothalamic tract (RHT) [16]. The SCN integrates and forwards the signals to the peripheral oscillators through transduction mediated by hormones, the autonomic nervous system, and the core body temperature [17]. The robust intercellular coupling of the SCN neurons underlies its exclusivity as a stable master oscillator [14]. However, peripheral tissues are also shown to gain circadian rhythmicity by direct response to particular zeitgebers, such as the daily feeding rhythm, without the interference of the SCN [14]. The synchronization of the central and peripheral clocks leads to cyclic behavioral and physiological responses [18].

**Figure 1:**
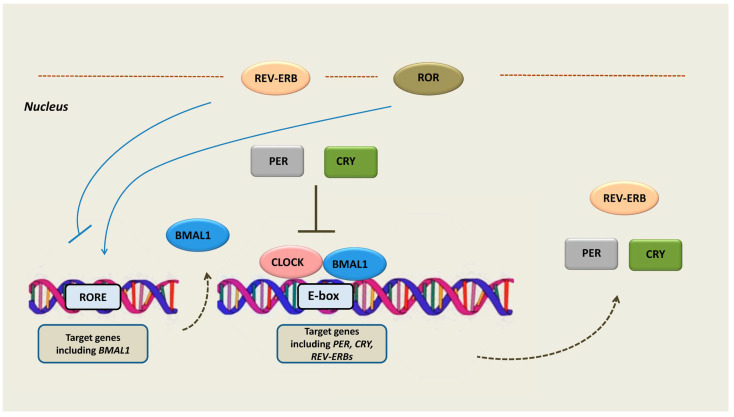
The molecular components of the cellular circadian clock. The circadian rhythm is generated by a main negative feedback loop in which the proteins PER and CRY translocate into the nucleus and inhibit the BMAL1 and CLOCK proteins, which are transcription activators of *PER* and *CRY*. Therefore, PER and CRY activation leads to their own transcription repression. An additional negative feedback loop consists of the expression activation of REV-ERBs by the CLOCK/BMAL1 complex. REV-ERBs act as transcription repressors while competing with RORs in binding to their response elements (ROREs) in target genes, including *BMAL1*. Abbreviations: PER = period, CRY = cryptochrome, BMAL1 = brain and muscle ARNT-like 1, CLOCK = circadian locomotor output cycles kaput, ROR = retinoid-related orphan receptors, REV-ERB = reverse erb, RORE = ROR-response elements.

At the cellular level, the circadian clock is a cell-autonomous sequence of expression, accumulation, and clearance of clock gene readouts. A set of clock genes expressed through positive and negative transcription–translation feedback loops (TTFLs) constitutes the basis of the circadian machinery: the molecular oscillator [18,19]. In particular, the circadian rhythm is generated by a main negative feedback loop in which the proteins period (PER 1/2/3) and cryptochrome (CRY 1/2) translocate into the nucleus and inhibit two activator proteins, the brain and muscle ARNT-like 1 (BMAL1) and circadian locomotor output cycles kaput (CLOCK). The latter two proteins are transcription factors that form complexes, also coupled with the neuronal PAS domain protein 2 (NPAS2). The complex binds to E2 boxes in the promoter of target genes, including *PER* and *CRY*, and enhances their expression [20]. Thus, by inhibiting the BMAL1/CLOCK complex, the PER and CRY proteins ultimately repress their own expression [20]. An additional negative feedback loop consists of the expression activation of REV-ERBs by the CLOCK/BMAL1 complex. REV-ERBs act as transcription repressors while competing with the transcription activators retinoid-related orphan receptors (RORs) in binding to their response elements (ROREs) in target genes, including *BMAL1* [21] (Figure 1).

Optimal alignment between the molecular components of the central and peripheral clocks, mainly through electrical and chemical signals, is essential to ensure the proper functioning of the individual systems. At the same time, increasing evidence supports the interference of circadian clock dysfunction in the pathogenesis of multiple human diseases [15]. Cell proliferation is a cellular process regulated by circadian rhythm, which characteristically shows asynchrony between normal and malignant tissues [22].

## 3. The Structure of REV-ERB Receptors

REV-ERBα was discovered in 1989 as a coding sequence on the reverse strand of the *c-erbα* gene, which encodes a thyroid hormone receptor [23,24]. The gene that encodes REV-ERBα is also known as the nuclear receptor subfamily 1 group D member 1 (*NR1D1*) gene. It is located on chromosome 17 and has one splice variant with eight exons. The identification of *REV-ERBβ* followed some years later [25]. Unlike *REV-ERBa*, *REV-ERBβ* has no significant reading frames in the opposite strand. More specifically, *REV-ERBβ*, also known as the nuclear subfamily 1 group D member 2 (*NR1D2)* gene, is located on chromosome 3 and has ten exons [26]. Alternatively to *NR1D1* and *NR1D2*, *REV-ERBα* and *REV-ERBβ* are also used to indicate the genes.

**Figure 2:**
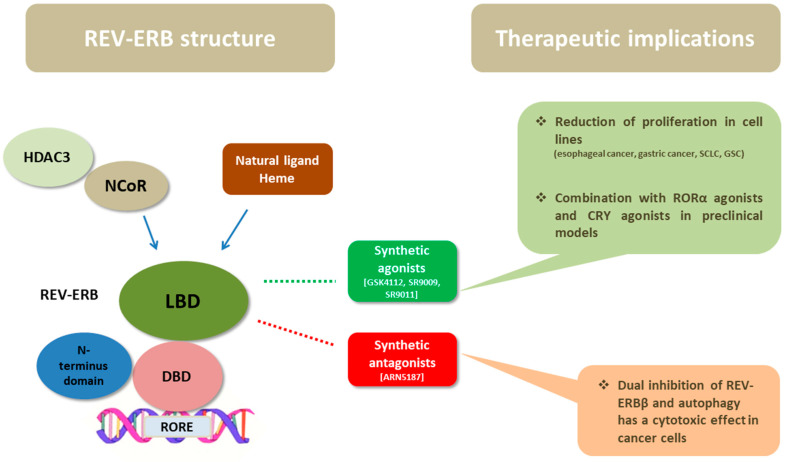
The structure of REV-ERBs consists of three main domains: the DBD, the LBD, and the N-terminus domain allowing activity modulation. Heme, the natural ligand of REV-ERBs binds to LBD and enhances the recruitment of NCoR and HDAC3. Their ligand-binding domain has fueled the development of synthetic ligands of REV-ERBs in order to serve as drugs. Synthetic agonists, namely, GSK4112, SR9009, and SR9011, have shown anticancer activity in preclinical studies of esophageal cancer, gastric cancer, SCLC, and glioblastoma stem cells; in some studies the combination of REV-ERB agonists with RORa or CRY agonists was promising. In addition, a synthetic ligand (ARN5187) with dual inhibitory effect in REV-ERBβ and autophagy demonstrated cytotoxic activity in cancer cells. Abbreviations: DBD = DNA-binding domain, LBD = ligand-binding domain, NCoR = nuclear receptor co-repressor, HDAC3 = histone deacetylase 3, REV-ERB = reverse erb, ROR = retinoid-related orphan receptors, CRY = cryptochrome, RORE = ROR-response elements, SCLC = small cell lung cancer, GSC = glioblastoma stem cells.

As members of the NR superfamily of proteins, REV-ERB receptors exhibit the canonical NR structure, which consists of a three-layered α-helical sandwich [27,28]. They have three main functional domains: a DNA-binding domain (DBD) and a ligand-binding domain (LBD) at the C-terminus, and an N-terminus domain allowing activity modulation (Figure 2). The DNA-binding domains of REV-ERBα and REV-ERBβ are almost identical, while their ligand-binding domains share 71% of the amino acid sequence identity [28]. An important feature of REV-ERBs is that they lack the carboxyl-terminal activation function 2 (AF2) at the C terminal end of the LBD, which usually recognizes co-activator protein partners that enhance transcription. This characteristic differentiates them from other NRs and it is the reason that they function as transcription repressors rather than activators [27,29,30,31]. The porphyrin heme has been identified as an endogenous ligand of both REV-ERBs, and it binds to the prototypical binding pocket of the LBD [32]. In addition to ligand binding, the activity of REV-ERBs is also regulated by post-translational modifications and protein–protein interactions [26].

## 4. Transcriptional Activity and Function of REV-ERBs

*REV-ERBs* are predominantly expressed in the brain and in major metabolic tissues, including the liver, skeletal muscles, and adipose tissue [33,34]. The two receptors function as ligand-dependent transcriptional repressors and display an overlapping pattern of expression [35,36].

As aforementioned, CLOCK and BMAL1 activate the transcription of *REV-ERBs* through E-boxes located within their promoter [37] (Figure 1). Subsequently, REV-ERBs recruit the nuclear receptor co-repressor (NCoR)/histone deacetylase 3 (HDAC3) complex and inhibit the gene expression of both the positive (*BMAL1* and *CLOCK/NPAS 2*) and the negative (*CRY* and *REV-ERB*) limb of the core clock [33]. It has been shown that REV-ERBs bind to DNA as either a monomer or homodimers, to specific sequences, namely, to an (A/G)GGTCA half-site with a 5′ AT-rich extension or to a direct repeat 2 element (AGGTCA sequence with a 2 bp spacer), respectively [1]. Interestingly, they compete with the transcriptional activators RORs at binding to ROREs in their target genes’ promoter and enhancer regions [34,38]. Τhe natural ligand, heme, binds reversibly to the LBD of REV-ERBs and enables efficient NCoR recruitment, therefore enhancing the repressive activity of REV-ERBs [28]. Mutations that hamper the binding of heme as well as variations in the intracellular heme’s levels modify the activity of REV-ERBs [32]. It should be noted that REV-ERBs do not interact functionally with a similar co-repressor, the silencing mediator of retinoic acid and thyroid hormone receptor (SMRT) [39].

The target genes of REV-ERBs are implicated in key cellular processes, mainly in metabolic pathways [39]. In addition to the regulation of the expression of *BMAL1* and *CLOCK*, REV-ERBs interact with uncoupling protein 1 (*Ucp1*), which regulates body temperature cycles, phosphoenolpyruvate carboxykinase (*PEPCK*), and glucose-6-phosphate (*G6P*), encoding key enzymes in gluconeogenesis, with the apolipoprotein (Apo) *ApoA1* and *ApoC-III*, which regulate cholesterol metabolism, the nuclear factor of the kappa light polypeptide gene enhancer in the B-cell inhibitor, alpha (*IkBα*), and other targets involved in inflammation [40,41]. Besides the circadian rhythm, various physiological processes and diseases have been associated with the activity of REV-ERBs, including cerebellar development, osteoarthritis, adipogenesis, and mitochondria biogenesis; nevertheless, their most well-established cellular function is the modulation of circadian rhythm and metabolic pathways [26]. Interestingly, dual depletion of *REV-ERBα* and *REV-ERBβ* by creating double-knockout mice led to profound disruption of circadian expression of the circadian clock and lipid homeostatic gene networks [38]. Nevertheless, evidence suggests that REV-ERBβ has a less critical role in maintaining the circadian rhythm compared to REV-ERBα since knockout studies of *REV-ERBβ* alone did not lead to major modifications of the circadian clock [33].

## 5. The Role of REV-ERB Receptors in Cancer Pathogenesis

Recently, several studies have investigated the putative role of REV-ERBs in carcinogenesis among different cancer types (Table 1). Hypothetically, it has been proposed that in the case of abnormal function of the circadian clock, the cells will take in excess energy for proliferation and metabolism, and the balance of autophagy may also be disrupted [22]. It has also been shown that REV-ERBα has a repressive function in cell proliferation and metabolism, which may be relevant during cancer pathogenesis [32]. Therefore, a series of studies discussed below have explored the expression and effects of REV-ERBs in clinical samples of cancerous tissues and preclinical models.

A recent study demonstrated that REV-ERBα was downregulated in human lung adenocarcinoma tissues, correlated with the primary tumor (T) and distant metastasis (M) stages, which indicate the anatomic extent of the tumor [42]. Further investigation in cell lines showed that the downregulation of REV-ERBα promotes the migration and proliferation of lung adenocarcinoma cells and also leads to increased expression levels of nuclear factor kappa-light-chain-enhancer of activated B cells (NF-κB), indicating a potentially implicated molecular pathway [42]. Interestingly, lung cancer and other malignancies are often associated with cachexia, an inflammatory response that causes the consuming of adipose tissue and skeletal muscle. In a preclinical model, it was reported that the existence of lung cancer in mice caused alterations in inflammatory and hormonal signaling that deregulate circadian pathways governing glucose and lipid metabolism in the liver [43]. Further investigation demonstrated how de novo glucose production in the liver is enhanced in a lung adenocarcinoma model. More specifically, it was shown that glucagon stimulates protein kinase A (PKA) signaling, leading to the destabilization of REV-ERBα and elevated hepatic glucose production in a model of lung cancer-associated cachexia [44].

Numerous studies have investigated the expression of REV-ERB receptors in gastrointestinal tumors. In a study of clinical samples from esophageal cancer, a comprehensive investigation of a set of genes involved in circadian rhythm was performed [45]. The results revealed significant downregulation of *CLOCK*, *PER1*, *PER2*, *PER3*, *CRY1*, *CRY2*, *REV-ERBα*, and *RORα* in cancer tissues compared with matched normal tissues. At the protein level, decreased levels of REV-ERBα were reported in esophageal cancer cell lines [45]. The cancer cell proliferation was reduced with the use of the REV-ERBα agonist SR9011 as well as with an RORα agonist [45]. Regarding gastric cancer, it was determined that REV-ERBα expression was decreased in tissues of patients with gastric cancer, which was correlated with poor differentiation (*p* = 0.009), T stage (*p* = 0.001), Tumor Node Metastasis (TMN) stage (*p* = 0.001), and lymph node metastasis (*p* = 0.007). In addition, decreased REV-ERBα expression was associated with poor prognosis (*p* < 0.05) [46]. In another study, an evaluation of the expression levels of RORα and REV-ERBα with immunohistochemistry and quantitative reverse transcription-polymerase chain reaction (qRT-PCR) revealed decreased expression in gastric cancer tissues compared with normal gastric tissues (*p* < 0.001) and the levels were associated with clinicopathological parameters, including histological grade, TNM stage, and preoperative carcinoembryonic antigen (CEA) levels [47]. Additionally, it has been shown that the knockdown of REV-ERBα in human gastric cancer cells significantly enhanced proliferation as well as glycolytic flux and the pentose phosphate pathway [48]. Treating gastric cancer cell lines with the REV-ERBα agonist, GSK4112, led to reduction of proliferation in a dose-dependent manner [48].

Furthermore, evidence derived from models of colorectal cancer revealed deregulated expression levels of clock genes, including REV-ERBs, in primary tumors and liver tissues. More specifically, in a murine model of colorectal cancer, downregulation of *REV-ERBα* and other clock genes was observed in primary tumors but not in surrounding colorectal tissue [49]. Interestingly, in the liver tissue of tumor-bearing mice, the clock gene rhythmicity was temporally shifted [50]. In addition, the expression levels of five clock genes (*REV-ERBα*, *PER1*, *PER2*, *BMAL1*, and *CRY1*) and three clock-controlled genes (D-Box binding PAR BZIP transcription factor or *DBP*, *p21*, and *WEE1*) were determined by qRT-PCR in colorectal liver metastases [50]. The results revealed the non-existence of 24 h oscillations for all clock and clock-controlled genes except *CRY1*. The researchers also investigated the expression levels of the above genes in the kidney. They suggested that the presence of a tumor leads to a phase shift of peripheral clocks in liver and kidney tissue, indicating that the cancer-associated deregulation of circadian clocks has a systematic nature [50].

Another study focusing on glioblastoma investigated the proliferation dependence on clock genes among different cell states of glioblastoma cells, namely, the glioblastoma stem cells (GSCs), differentiated glioblastoma cells, and non-malignant brain cells [51]. The GSCs only exhibited a strong dependence on clock-related transcription factors. The treatment with the small-molecule agonists of REV-ERBs SR9011 and SR9009 led to a reduction in the GSC proliferation rate [51]. Noteworthily, the combination of SR9011 with the CRY agonist KL001 augmented the disruption of GSC growth [51]. Furthermore, whether the clock gene expression was associated with viable cell functions was investigated in glioblastoma cell lines [52]. In arrested cultures, mRNAs for clock (*PER1*, *REV-ERBα*) genes followed circadian rhythmicity as opposed to proliferating cells, where gene expression rhythms were lost or their periodicity shortened. Nevertheless, other metabolic-related genes, such as the glycerophospholipid (GPL)-synthesizing enzyme genes, maintained their fluctuation in proliferating cells with a similar periodicity as under arrest. The study indicated that an intrinsic metabolic clock continues to function in proliferating cells, potentially highlighting differential states of tumor suitability for more efficient, time-dependent chemotherapy [52].

A set of clock genes, including *REV-ERBα* and *REV-ERBβ*, was investigated in cervical cancer tissues [45]. Significant downregulation of the core circadian clock genes, *CLOCK*, *BMAL1*, *PER1*, *CRY1*, *REV-ERBα*, and *RORα*, was found in cervical cancer tissue specimens and in high-grade squamous intraepithelial lesions compared to the normal cervical epithelium [45].

Since clock genes are important contributors to normal adrenal function, their role in adrenal tumorigenesis has also been explored in clinical samples of normal, benign, and cancerous adrenal tissue [53]. Significant downregulation of *PER1*, *CRY1*, and *REV-ERB* genes compared with their normal tissue was demonstrated in cortisol-secreting adenomas. A significant upregulation of *CRY1* and *PER1* and downregulation of *BMAL1*, *RORα*, and *REV-ERB* compared with normal adrenal tissue were observed in adrenocortical carcinomas. In particular, *REV-ERBβ* was downregulated in adrenocortical carcinomas compared to benign cortisol-secreting adenomas [53].

**Table 1:** Studies reporting the expression of REV-ERBs in clinical samples of cancer tissues.

Author (Year)	Tumor Type	Main Findings	[Ref.]
Zhang et al. (2022)	NSCLC (adenocarcinoma)	REV-ERBα downregulation; correlation with T,M stage	[42]
Verlande et al. (2021)	NSCLC (adenocarcinoma)	REV-ERBα downregulation in liver tissue; involved in lung cancer-associated cachexia	[44]
van de Watt et al. (2020)	Esophageal cancer	REV-ERBα downregulation; reduced cancer cell proliferation after REV-ERBa agonist	[45]
	Cervical cancer	REV-ERBα downregulated in cervical cancer and high-grade squamous intraepithelial lesions	[45]
Wang et al. (2018)	Gastric cancer	REV-ERBα downregulation; correlation with TNM; correlation with poor survival	[46]
Wang et al. (2021)	Gastric cancer	REV-ERBα downregulation; correlation with TNM stage, histological grade, CEA	[47]
Tao et al. (2019)	Gastric cancer	Knockdown of REV-ERBα leads to increased proliferation; REV-ERBα agonist restores the effect	[48]
Sotak et al. (2013)	Colorectal cancer	REV-ERBα downregulation in CRC tissue; temporal shift of circadian rhythm in liver tissue	[49]
Huisman et al. (2015)	Colorectal cancer	REV-ERBα downregulation in liver metastases	[51]
Wagner et al. (2019)	Glioblastoma	Glioblastoma stem cells exhibit growth dependence on circadian rhythm	[52]
Angelousi et al. (2020)	Adrenal benign and malignant tumors	REV-ERBs downregulation in cortisol-secreting adenomas and adrenocortical carcinomas	[53]

Abbreviations: TNM = Tumor Node Metastasis; CEA = Carcinoembryonic Antigen.

## 6. Therapeutic Considerations

REV-ERBs act as transcription repressors and their activation is enhanced by binding their natural ligand, heme, in the LBD of their molecule. In the last few years, there has been considerable research interest, chiefly in the field of metabolic disorders, in identifying novel synthetic modulators of REV-ERBs to serve as drugs [54,55] (Figure 2). Synthetic agonists of REV-ERBs have been developed chiefly based on measuring the interaction between REV-ERBα and its co-repressor NCoR1 [54,56].

In the field of cancer therapeutics, an intriguing hypothesis holds that pharmacological modulation of the circadian machinery may be an effective therapeutic strategy. A specific lethal effect of two REV-ERBs agonists, SR9009 and SR9011, against cancer cells and oncogene-induced senescent cells but not against normal cells has been reported [57]. The activity of SR9009 and SR9011 is effective against several oncogenic pathways and is maintained in the absence of p53 and under hypoxic conditions [57]. Further investigation revealed that the impairment of cancer cells was caused by the induction of apoptosis after treatment with SR9009 and SR9011 and by inhibition of autophagy, which is usually hyperactivated in the cancer cells because of their high metabolic demands [57,58].

The REV-ERB synthetic agonist SR9009 was recently used in a preclinical model of chemosensitive and chemoresistant small cell lung cancer (SCLC), a typically rapidly dividing tumor type [59]. The study reported that SR9009 exhibits potent anticancer activity in chemosensitive and chemoresistant SCLC cells. The core autophagy gene autophagy-related 5 (*Atg5*) was identified as a direct downstream target of REV-ERBα, and it was suppressed after treatment with SR9009 [59].

Another interesting potential therapeutic implication of the REV-ERB agonist SR9011 was investigated in breast cancer models based on the molecule’s structural similarity with the v-erb-b2 avian erythroblastic leukemia viral oncogene homolog 2 (*ERBB2)* gene, also known as human epidermal growth factor receptor 2 (*HER2*), which is often mutated in breast cancer [60]. The therapeutic approach to breast cancer typically differs according to hormone receptor and HER2 status. This study was conducted on a range of estrogen receptor (ER)-positive, ER(−), HER2(+), HER2(−), and triple-negative breast cancer cell lines. The results demonstrated that SR9011 suppressed the proliferation of the breast cancer cell lines regardless of their ER or HER2 status. More specifically, the treatment with the REV-ERB agonist SR9011 resulted in a significant decrease in cyclin A2 levels of cancer cells, leading to a reduction in cell viability in a dose-dependent manner and independently from the ER or HER2 status [60].

Finally, although most therapeutic implications involve using REV-ERB agonists, a special focus should be placed on the potential use of REV-ERBβ inhibitors in certain cases. Importantly, REV-ERBβ is not a significant contributor to cancer cell viability per se, which is evident from studies of its genetic and pharmacological inhibition that do not significantly impact cell death or proliferation. However, this nuclear receptor has a critical and unexpected role in supporting cancer cell viability when autophagy is compromised [61]. Indeed, the dual inhibition of REV-ERBβ and autophagy has been investigated and seems to be an effective strategy for eliciting cytotoxicity in cancer cells [62]. It has been shown that REV-ERBβ is a determinant of sensitivity to chloroquine, a clinically relevant agent that suppresses autophagy. Interestingly, a dual synthetic ligand of REV-ERBβ was developed (ARN5187) with a dual inhibitory effect on REV-ERB transcriptional activity and autophagy and it showed strong cytotoxic activity in preclinical investigation [62].

## 7. Discussion

REV-ERBs are peripheral pacemakers of circadian rhythm and function as transcription repressors of several target genes primarily implicated in metabolic pathways [1]. Research on their expression and role in cancer pathogenesis reveals that in the majority of studies the two receptors are downregulated in cancerous tissues; their downregulation and, in particular, that of REV-ERBα are often associated with increased cell proliferation in cell lines, and the use of synthetic agonists seems to restore the cell viability in certain cases [31].

Despite the pharmacological effects of REV-ERB ligands in preclinical studies, there are no advances toward their translation to clinical development across all relevant therapeutic fields, including cancer. This difficulty implies that certain challenges associated with the drug development of REV-ERB ligands exist, potentially related to drug safety and bioavailability [31]. A potential limiting factor may be the expression of REV-ERBs in many tissues, which shapes broad actions but may be associated with adverse events [63].

Additionally, even though putative REV-ERB agonists have been used in numerous preclinical studies, the pathways that mediate their effects have not been clearly proven and may be partially REV-ERB-independent. Intriguingly, thorough research using a mouse model of conditional genetic deletion of REV-ERBα and REV-ERBβ demonstrated that the effects of SR9009 on proliferation, metabolism, and gene expression cannot be attributed exclusively to its regulation of cellular REV-ERB activity [64]. The researchers underlined that the pleiotropic effects of SR9009 and related compounds on cell proliferation, metabolism, and gene expression are mediated by pathways that remain to be clarified, and so they should not be surrogates of REV-ERB activity [64]. In line with this finding, a recent study that used SR9009 in prostate cancer cell lines revealed cytotoxic effects; however, further investigation did not prove involvement of REV-ERBs but rather activation of another NR, the liver X receptor α (LXRα) [65].

It has been suggested that the “engagement” of the circadian rhythm for increasing the activity of existing anticancer drugs may be a more effective approach compared to targeting the molecular clock alone, given that in most cases and based on current evidence, the aberrations of the circadian rhythm do not seem to be a central driver event during carcinogenesis but an additional, non-negligible contributor [61]. In this scenario, agents such as those targeting the REV-ERB receptors might be suitable for combination approaches, and therefore, further investigation of the appropriate safe and effective combinations is greatly anticipated [63].

Finally, an exciting consideration is the possibility of harnessing the modulation of circadian machinery in order to optimize the effects of existing anticancer therapies. This approach could have two potential aspects. The first is to modulate the delivery and metabolism of anticancer drugs in order to maximize the therapeutic potential and minimize the therapy-related adverse events [66,67]. In fact, it has long been known that the different administration time of cisplatin and doxorubicin in patients with ovarian cancer is associated with toxicity [68]. Similar findings were reported in cohorts of patients with colorectal cancer who were treated with oxaliplatin, fluorouracil, and folinic acid [69]. It should also be noted that interpatient heterogeneity exists with regard to the circadian rhythm; therefore, a personalized approach might be needed [63,70]. This evidence suggests that there might be a potential benefit from attempting to “chrono-modulate” certain drugs, aiming at their optimal delivery and metabolism patterns [63]. The second aspect is based on the fact that the majority of existing FDA-approved drugs (regarding any disease) target proteins that exhibit circadian rhythmicity of expression [71]. It has been proposed that aligning the clock between the target gene and the delivered drug might augment the therapeutic benefit [63]. This consideration has mainly been explored in other fields, such as the anti-inflammatory and glucose-regulating drugs, but it opens up the avenue for potential applications in different therapeutic areas [70].

## 8. Conclusions

REV-ERB receptors are nuclear receptors that function as circadian clock-associated transcription repressors, being part of an important transcription–translation feedback loop along with other essential clock genes. Recent studies have explored their role in cancer pathogenesis. In most of the studies, they are found to be downregulated in human cancerous tissues and preclinical cancer models, and their downregulation is associated with cell proliferation in cancer cell lines. The pharmacological restoration of their effects is feasible with synthetic agonists, which have been explored in preclinical studies but with scarce data. Drug development and clinical translation in this field are challenging and require better characterization of REV-ERB deregulation and association with specific malignant phenotypes. Moreover, targeting the REV-ERB receptors could be exploited in order to “chrono-modulate” other anticancer drugs and optimize their outcomes. There is a need for further investigation on the effect of the REV-ERB-induced circadian rhythm deregulation on carcinogenesis and cancer-related systemic effects such as cachexia in order to address the potential of relevant therapeutic implications.

## Data Availability

No new data were created or analyzed in this study.
